# Mental Nursing
*The substance of two lectures delivered to the nurses of the West London Hospital by special request of the Matron and the Dean of the Post-Graduate College.


**Published:** 1919-11-15

**Authors:** Robert Armstrong-Jones

**Affiliations:** Lecturer on Mental Diseases to St. Bartholomew's Hospita. Formerly Medical Superintendent of the L.C.C. Asylum, Claybury.


### MENTAL NURSING.*

BY SIR ROBERT ARMSTRONG-JONES, M.D., F.R.C.P., F.R.C.S., C.B.E.

Lecturer on Mental Diseases to St. Bartholomew's Hospita. Formerly Medical Superintendent of the L.C.C. Asylum, Claybury.

In no disease is the help of the doctor and the nurse so essential as or more appreciated than in serious mental illness, and a good nurse is of incalculable help at this crisis.

The ideal qualities of a good mental nurse are the rare possession of but a few, because she should have in combination the skill of the hospital nurse—which are qualities of the head—with the sympathy, the firmness, the disciplined mind, the tact, and the good settled principles of the mental nurse—which are qualities more of the heart, for she has to set an example to her patients of right conduct, she must be courteous and loyal, she must be able to instil into her patients habits of industry, punctuality, order, neatness, and especially of cleanliness, because her personal example and influence are elements in the treatment that depends so much upon the discipline of re-education and encouragement.

To the mental nurse the satisfaction of doing right must often be the only moral reward, for the mental nurse is called upon to treat patients who are detained against their will, who are, in consequence, suspicious of those around and about them, who literally chafe at the compulsory restraint to which they are subjected; and they are therefore resentful of, rather than grateful for, anything that is done for their benefit and welfare. They may often be rude to the nurse and the doctor, but neither of these can retaliate or show any feeling of hostility or anger in return, because the insane are not responsible. They are not punished for any shortcomings in the matter of gratitude or courtesy, and they are not blamed for any errors of conduct. The atmosphere and tone of the ward in the mental hospital is therefore totally different in most respects from that which is the rule in the ordinary sick hospital.

### A Comparison.

In the latter, as you know, the patient is expected to respect the rules and to conform to them; he is under the obligation to do what he is told, to be orderly in his conduct, and to entertain and to show a due regard to those in authority, and this under the penalty of dismissal. Usually, also, when he is well enough to get out of bed, he is discharged home, but in the mental hospital rule and order are maintained by the force of example rather than by admonition and correction. In the mental hospital the nurse has often to put up with personal ridicule, with gibes and sneers, with angry responses to kind inquiries, and she may have to bear various exhibitions of indiscipline and lack of control, perhaps amounting to violence, but which can only be checked by the influence of a good example. These trying indiscretions of a mental patient must never be brought up against the patient or regarded as a prejudice to his interests. The mental nurse has, further, to exercise the Hippocratic virtue of *reticence*; for if she hears anything that is not right she has to be silent and not bring it up against him as a reflection or an accusation, counting what has been said as unsaid. As compared with the hospital nurse, she has to win her control over the mental patient by her kindness, forbearance, and her own self-discipline, and, unlike the control of the ordinary sick nurse, the mental nurse cannot command it, she can only claim it by force of example, which is naturally an exceedingly slow process. In no other position in life are tact and wisdom—characters that are inborn, yet which may be strengthened and developed—so requisite as in dealing with mental patients, and under no other circumstances does the soft answer which turneth away wrath count for more power and real influence for good than in the care and control of mental diseases. Self-control, self-direction, self-culture, and reticence are, therefore, the essential qualities of the mental nurse.

### The Aim of Treatment.

In a large mental hospital the aim of treatment is (1) to promote recovery; (2) in cases where recovery does not take place, to make the surroundings as favourable, healthy, and comfortable for the patients as is possible; and (3) to secure that all the patients shall be kept under due observation; and it is thus necessary that the nurse in an institution should make herself familiar with its arrangements and regulations, and carry them out in a loyal manner. She should study the rules presented to her, and obey them without argument, as the rules suggest how the institution is best run efficiently and economically, and if these are intelligently observed the duties of the day will be well done and punctually carried out, for there are definite times for meals, bathing, exercise, rising, and retiring to bed. It will be a discovery to the new nurse to find that certain doors in the mental hospital are always kept locked when closed, that although there may be a considerable amount of liberty allowed to patients in some wards, in other wards the doors are carefully secured, and it is a rule always to lock a door which is not meant to be left open. The mental nurse will find that there are certain duties imposed upon her by the Lunacy Laws, and in particular as to neglect or harsh treatment, which is guarded against under a penalty, and although it may be difficult to define neglect, it is interpreted to be any act by which the patient suffers. The chief treatment of the insane during the early years of last century was through restraint, which means that something was applied by which the patient was prevented from moving the body or a limb; but to-day there is no mental institution, public or private, in the country which possesses a single implement of restraint. The treatment of mental diseases has greatly improved, and whenever it is now necessary to use restraint there is a legal obligation to report in writing fully the means employed, the duration of its application, and the reason for its use, which is to-day only employed for surgical reasons. It is also laid down in the rules of institutions that no seclusion may be applied to any patient unless the same is reported in writing, seclusion being the enforced isolation of a patient by locking the door of a room or closing it in a manner that the patient may not be able to open it from within, and this usually between the hours of seven in the morning to seven in the evening, during which hours it is naturally expected that a patient who is not infirm will be up and about. Very strict rules must be observed about letters written by mental patients. They must be forwarded unopened

* The substance of two lectures delivered to the nurses of the West London Hospital by special request of the Matron and the Dean of the Post-Graduate College.

**Mental Nursing**—(*continued*).

when addressed to certain persons, and this again under a penalty. Another penalty may be imposed upon a nurse or a person who assists a patient to escape, or upon a nurse who commits an assault on a patient. The treatment of the insane, formerly both dangerous and cruel, and even desperate and barbarous, is to-day humane and generous, philanthropic and scientific.

One of the most trying forms of mental disease that a nurse may be called upon to treat is the depressed variety, in which the patient may refuse food and in whom there is a tendency to suicide. The utmost care must always be taken, especially when nursing a "private case," to avoid giving an opportunity for the patient to do harm to himself or herself. All strings, all cutting instruments such as razors or scissors, all access to water or to fires must be guarded, and all ladders or windows and staircases must be protected. Suicidally disposed patients must be constantly and continually supervised, and the supervision in these cases must be direct, for they must not be permitted to wander about or to get out of sight. In some instances the supervision is so direct that one nurse signs a caution paper when handing the care of the patient to another nurse. The other kind of mental patient is of the excited or agitated type, and in nursing these cases great care must be taken to avoid struggles, and a nurse should never attempt to control such a patient single-handed. It is better to leave an excited patient alone than to run the risk of incurring bruising or fractured ribs.

The different forms of mental case that a nurse may be called upon to treat may be complicated and be said to present nervous symptoms of about six kinds: (1) Motor symptoms, such as those with tremors, or spasms, paralysis, or a want of co-ordination; (2) sensory symptoms on one or other side, such as loss of pain or sensation, and care must be exercised in the use of the hot-water bottle for these patients; (3) those with affections of the special senses or who have delusions in regard to them; (4) those in whom the reflexes are affected, such as cases of locomotor ataxia or general paralysis, or those who are suffering from the chronic effects of alcohol; (5) patients who have nervous disturbances of nutrition, such as wasting of muscles or disorders of the joints or bones; and (6) those who suffer from affections of the bladder or the rectum through incontinence, which indicate serious dementia or loss of mind-power. The mental symptoms presented by the patient are described as either illusions when there is an actual object present, hallucination without an object, as, for instance, when the wind is whistling through the keyhole and is interpreted as the voice of God it is an illusion; but when there is nothing heard and the patient still insists upon the statement, it is a hallucination, and the latter indicates a deeper reduction of mental disease. A delusion implies a false belief which cannot be removed by any appeal to reason, as when the patient states he is the Almighty, or his enemies have put ground glass in his food, or his wife has tried to poison him, or that he is being electrified against his will. These are delusions, and it is most necessary that the nurse should not discuss them with an irritable patient, but should listen and attend sympathetically, and if asked for an explanation should reply that probably his brain had played tricks with him or that his memory had been false. In all dealings with a mental patient, it should be on the assumption that he is an ordinary sane person and that he should be treated as a reasonable being.

In the treatment of individual cases the nurse's chief effort should be to endeavour to gain the patient's confidence by kindness and sympathy, to explain any misapprehension that he may entertain, and to act the real "friend" to him. It is essential that the nurse should be cheerful and hopeful, which is the best attitude to combat distressing fancies. If he can be interested in things around him by inducing him to engage in some active work or amusement recovery has probably commenced, and to find a healthy outlet which may be congenial is the best diversion for the turbulent and noisy. Most acts in conduct have an emotional aspect, and if by suggestion the emotion can be re-induced which may have helped the mental breakdown and he is then reasoned with, then a restoration may be effected. The emotions of love and hate and fear are often related to a mental breakdown as etiological or causative factors. A tactful nurse thinks for her patient, and she has to use her own mind for his even in all the routine affairs of daily life.

There are four conditions which demand special attention from the mental nurse—(*a*) homicidal attempts, and notably the epileptic or the alcoholic is the essentially impulsive patient. He is often prompted by delusions or hallucinations to unreasonable and unprovoked assaults upon others, and he must have ceaseless watching, all cutting instruments and all keys being kept from him. (*b*) Refusal of food, which must be met by active nursing and the forcible administration of easily assimilable nourishment. This is imperative, and by means of the nasal tube if necessary, but the nurse should not attempt to carry out forcible feeding. (*c*) Retention, which should be closely watched and looked for, as owing to the insensitiveness which is associated with serious cases of mental disease the bladder may be ruptured. (*d*) Sleep must be obtained at all costs and correct reports as to this are most necessary in order to help the doctor's treatment.

In the insane the mental condition is largely affected by the state of the body, and this is influenced by the ordinary conditions which normally promote bodily welfare; such are attention to ventilation, cleanliness, warmth, and sufficient food, regular open-air exercise, and sleep with sufficient bedding at night. As taking food is under the will, so to a less extent is getting rid of it, and as the will is lacking in mental cases so they suffer from constipation. The patient must take aperients, for constipation is the dominating evil in mental cases, and he must be kept clean, especially the teeth and the skin, and the good condition of the digestion is imperative; although normal digestion occurs apart from the influence of the will, it is nevertheless under brain control, and if the food is eaten too quickly or without proper mastication, indigestion follows. The nurse should see that food is not removed with undue haste and that the patient should receive the proper amount of nourishment. The private nurse should report as to whatever enters the patient's body and whatever comes away, as also any change in the mental state. The sleep, pulse, respiration, and temperature must be reported, as also any struggles. The time passed in the open air should also be in the report, and the patient's weight must be taken weekly and recorded, as it is often the best indication of the result of treatment. Our last advice is that the mental nurse should be an abstainer from drink whilst in charge of the patient. The distortion of facts by the patient, the suspicion of relatives, and the gossip of outsiders should rouse the mental nurse to realise her or his position of trust and responsibility; but her character will carry her through all difficulties; yet she should be watchful, thoughtful, and self-reliant, and no joy is equal to the pleasure of seeing a mental patient with whom one has troubled emerging from the dark cloud into the light.

